# *Abiotrophia defectiva* causing infectious crystalline keratopathy and corneal ulcer after penetrating keratoplasty: a case report

**DOI:** 10.1186/1869-5760-3-20

**Published:** 2013-01-25

**Authors:** Yannis M Paulus, Glenn C Cockerham

**Affiliations:** 1 Department of Ophthalmology, Stanford University, Palo Alto, CA 94305, USA; 2 Ophthalmology Section, Veterans Administration Palo Alto Health Care System, 3801 Miranda Avenue, Palo Alto, CA, 94304, USA; 3 Department of Pathology, Stanford University School of Medicine, 300 Pasteur Drive, Stanford, CA 94305-5324, USA; 4 Byers Eye Institute, Stanford Hospital and Clinics, 2452 Watson Court, Palo Alto, Stanford, CA, 94303-5353, USA

**Keywords:** *Abiotrophia defectiva*, corneal ulcer, fastidious streptococcus, infectious crystalline keratopathy, nutritionally deficient streptococcus, penetrating keratoplasty

## Abstract

**Background:**

Infectious crystalline keratopathy is commonly caused by *Streptococcus viridans* and other gram positive organisms. We present the first case of infectious crystalline keratopathy that developed into a corneal ulcer and grew *Abiotrophia defectiva* which responded well to topical and systemic antimicrobial therapy and did not require re-grafting. A 78-year-old man underwent penetrating keratoplasty for pseudophakic bullous keratopathy. He presented 1.5 years later with infectious crystalline keratopathy which progressed to a corneal ulcer. The patient received topical fortified vancomycin and moxifloxacin, as well as oral moxifloxacin.

**Findings:**

The corneal ulcer base was cultured and grew *A*. *defectiva*, or nutritionally deficient streptococcus. Complete resolution of the corneal infiltrates was obtained within three months.

**Conclusions:**

Nutritionally deficient streptococcus has been implicated in numerous human diseases, including endocarditis, and is increasingly being recognized as an important pathogen. This represents the second reported case of *A*. *defectiva* causing infectious crystalline keratopathy in humans and the first case of *A*. *defectiva* successfully treated with antibiotics. This case shows that aggressive antibiotic therapy can be effective in *A*. *defectiva*-associated infectious crystalline keratopathy.

## Findings

### Introduction

Infectious crystalline keratopathy (ICK) was first described in 1983 and is a rare complication following corneal transplantation and chronic inflammation [1]. It is characterized by the formation of branching and crystalline-appearing opacities deep in the corneal stroma that are formed from localized invasion of microorganisms without adjacent inflammation. *Streptococcus viridans* is the most common causative organism, but other common pathogens include *Staphylococcus epidermidus*, *Corynebacterium*, *Pseudomonas aeruginosa*, and fungi [2]. We present a case of a man with infectious crystalline keratopathy that developed into a corneal ulcer and grew *Abiotrophia defectiva*, or nutritionally deficient streptococcus (NDS), which responded well to topical and systemic antimicrobial therapy and did not require re-grafting. An IRB waiver was granted, and consent was obtained from the patient. All research was performed in accordance with the Declaration of Helsinki and all local, regional, and national laws.

### Case report

A 78-year-old man with a history of a ruptured globe, anterior chamber intraocular lens, and pseudophakic bullous keratopathy presented 1.5 years status-post penetrating keratoplasty on prednisolone 1% daily. The patient was asymptomatic, lived next to horses, and had a penicillin allergy. Examination demonstrated a visual acuity of 20/400 with pinhole to 20/150, which was stable from three months prior. The graft was well-centered with five remaining interrupted sutures and a running suture, all of which were buried. A crystalline branching pattern extended from the graft-host interface at 9 o'clock centrally at approximately 60% depth. The epithelium was intact and the anterior chamber quiet. Central corneal thickness was 533 microns. He was started on moxifloxacin ophthalmic and vancomycin fortified for ICK. Prednisolone was discontinued.

**Figure 1:**
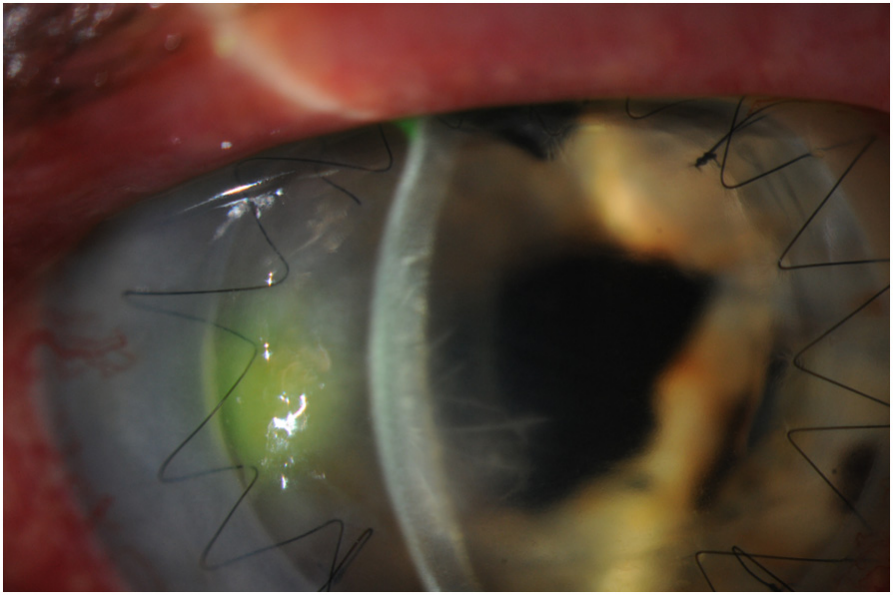
**Slit lamp photograph one week after initial presentation. **An epithelial defect corneal ulcer 2.5 mm in size overlying the area of crystalline keratopathy, which extends 5.4 mm into the transplant, with keratic precipitates and anterior chamber reaction.

One week later, the patient was unable to get his medications and reported increasing pain and redness. A new 2.5 mm epithelial defect was present along with moderate anterior chamber inflammation and large keratic precipitates (Figure 1).

### Results

**Figure 2:**
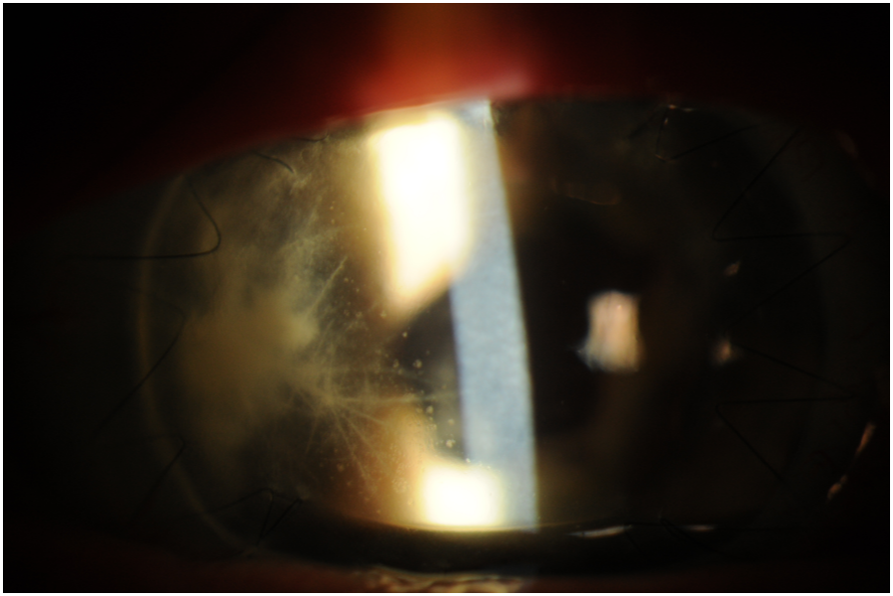
**Slit lamp photograph two weeks after initial presentation. **This shows the resolution of the epithelial defect. The intralamellar branches remain.

**Figure 3:**
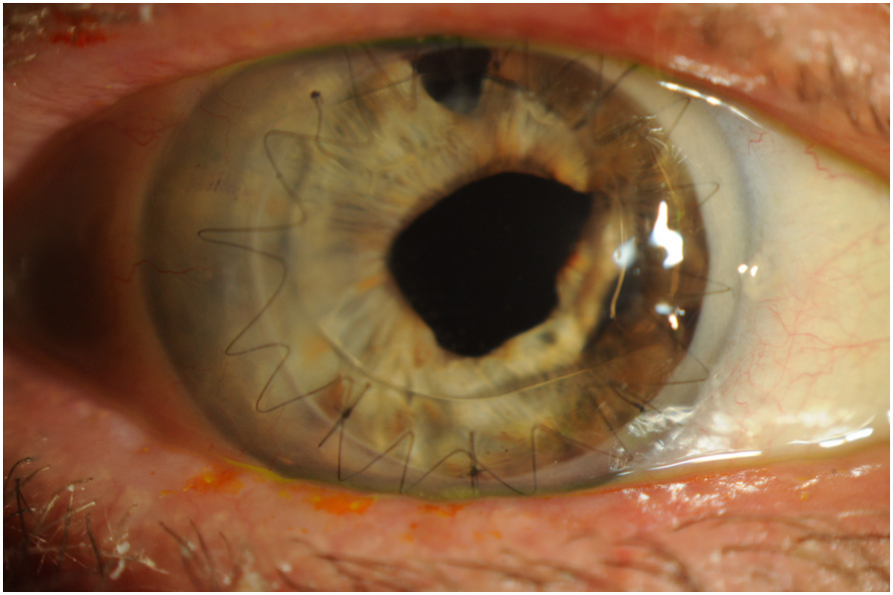
**Slit lamp photographs taken three months after initial presentation. **This shows the resolution of the infectious crystalline keratopathy, corneal stromal infiltrate, anterior chamber reaction, and conjunctival injection with no signs of graft rejection.

Corneal cultures were obtained. Fortified vancomycin, moxifloxacin, and natamycin were begun hourly, and ciprofloxacin was given orally. The patient's symptoms improved over the next week, and the epithelial defect resolved (Figure 2). Over one month, the crystals had disappeared and the patient was asymptomatic. After three months, the patient has a visual acuity pinholing to 20/80, resolution of the crystals, and no signs of corneal graft rejection (Figure 3).

The corneal culture initially reported a few gram positive rods. Subsequent speciation showed two colonies of streptococcus, nutritionally deficient (fastidious), or *A*. *defectiva*. This diagnosis was reached by colony growth on chocolate agar plates but not blood agar, a distinctive butterscotch-caramel odor, satellite testing where the organism was streaked on plates of *Staphylococcus aureus* and noted to only grow adjacent to the *S*. *aureus*, and biochemical analysis using the API Rapid Strep panel (bioMerieux, Marcy l'Etoile, France) and Vitek GP (bioMerieux Vitek Inc., MO, USA) using a biochemical algorithm. Prednisolone acetate 1% daily was restarted, and the patient remains on this medication.

### Discussion

Infectious crystalline keratopathy is a rare complication typically following penetrating keratoplasty, but it can occur in an ungrafted cornea in patients with herpes simplex, herpes zoster, *Acanthamoeba*, or local anesthetic abuse [3,4]. Three cases have been reported of patients not on topical steroids, but instead systemically immunosuppressed [5]. Many organisms have been isolated in cases of ICK, but the most common are gram positive aerobic streptococci which have been reported in 42% of cases, of which *S*. *viridans* is the most common [2]. Another 12% of cases are reported with staphylococci as the organism isolated, including *Staphylococcus aureus* and *haemolyticus*. Fungi have been implicated in 8% of cases, including *Candida tropicalis*, *albicans*, and *parapsilosis*, and *Alternaria*. Additional organisms that have been isolated include *Mycobacterium fortuitum*, *Peptostreptococcus*, *Corynebacterium*, *Pseudomonas*, and *Acanthamoeba*. Often, multiple organisms are isolated.

NDS, also called nutritionally variant streptococcus or fastidious streptococcus or the *Abiotrophia* genus, has been demonstrated to be a pathogenic organism in humans since 1961 and was first reported in cases of subacute bacterial endocarditis [6]. NDS requires a thiol-containing compound (e.g., pyridoxal) and vitamin B_6_ for growth. Typical culture media do not contain these compounds, and thus NDS are often difficult to grow. NDS is often seen as satellite lesions around other bacterial organisms that secrete pyridoxal, such as *Staphylococci* or *Haemophilus influenzae*. NDS has primarily been reported in human cases of subacute bacterial endocarditis. It has also been implicated in pancreatic abscesses, brain abscesses, prosthetic infections, osteomyelitis, vaginal infections, otitis externa, and superficial skin wounds. NDS are normal inhabitants of the human oral and respiratory flora, and respond poorly to conventional antibiotics. The *Abiotrophia* genus includes four species: *Abiotrophia defectiva*, *adiacens*, *balaenopterae*, and *elegans*. *Abiotrophia adiacens* has been implicated in infectious crystalline keratopathy, endophthalmitis, and neonatal conjunctivitis [7].

The first paper reporting NDS as a causative agent of corneal ulcers was from the equine literature. A retrospective review of 259 eyes of corneal ulcers in horses in the 1980s in California showed 35 isolates of NDS, or 13.5% of their cases [8]. This study also performed antimicrobial sensitivity analysis and showed that only two cases were resistant to penicillin. All the others were pan-sensitive to antibiotics, and thus the authors recommended that routine sensitivities not be performed on NDS growth.

A case series of four cases of infectious crystalline keratopathy in humans associated with NDS was published in 1990 [9]. In this case series, additional organisms were cultured in each case, and thus the causative organism was unclear. One patient was status-post penetrating keratoplasty who underwent repeat penetrating keratoplasty for the infectious crystalline keratopathy, which grew *S*. *viridans*, *S*. *aureus*, and NDS. Another patient was an aphakic extended soft contact lens wearer on fluorometholone who grew *S*. *viridans* and NDS. Another grew *S*. *aureus* and NDS, and the last was a case of fungal keratitis, who also grew NDS.

The first case of *A*. *defectiva* infectious crystalline keratopathy was reported in 2010 from France in an 83-year-old female who was 10 months status-post penetrating keratoplasty for pseudophakic bullous keratopathy [10]. The patient developed infectious crystalline keratopathy which progressed despite topical ciprofloxacin and vancomycin, and thus underwent a repeat penetrating keratoplasty, which showed *A*. *defectiva*.

### Conclusion

This case report demonstrates the second case of *A*. *defectiva* causing infectious crystalline keratopathy in humans and the first case successfully treated with antibiotics without need for re-grafting. Nutritionally deficient streptococcus has been implicated in numerous human diseases, including endocarditis, and is increasingly being recognized as an important pathogen as our ability to culture this pathogen improves and, thus, should be considered as a potential causative organism in corneal ulcers and infectious crystalline keratopathy. Our case demonstrates that aggressive antibiotic therapy with both topical-fortified antibiotics and systemic oral antibiotics can be effective in *A*. *defectiva*-associated infectious crystalline keratopathy.

## Competing interests

The authors declare that they have no competing interests.

## Authors’ contributions

YP performed the clinical examinations, cultured the corneal ulcer base of the patient, performed a literature review, discussed the case with a microbiologist, and drafted and edited the manuscript. GC performed the clinical examinations and surgical procedures, interpreted the histopathology, obtained photographs, and participated in drafting the manuscript. All authors read and approved the final manuscript.
